# Clinical value of docetaxel plus cisplatin (TP) induction chemotherapy followed by TP concurrent chemoradiotherapy in locoregionally advanced nasopharyngeal carcinoma

**DOI:** 10.7150/jca.49944

**Published:** 2021-01-01

**Authors:** Hao-Yun Tao, Ze-Jiang Zhan, Wen-Ze Qiu, Kai Liao, Ya-Wei Yuan, Tai-Ze Yuan, Rong-Hui Zheng

**Affiliations:** 1 Department of Radiation Oncology, Affiliated Cancer Hospital & Institute of Guangzhou Medical University, Guangzhou, 510090, Guangdong, P. R. China.; 2 Department of Radiation Oncology, Guangzhou Concord Cancer Center, Guangzhou, 510045, Guangdong, P. R. China.

**Keywords:** Nasopharyngeal carcinoma, concurrent chemoradiotherapy, propensity score matching, docetaxel, cisplatin

## Abstract

**Objective**: To investigate the clinical value of induction chemotherapy (IC) with docetaxel plus cisplatin (TP) followed by concurrent chemoradiotherapy (CCRT) with TP in locoregionally advanced nasopharyngeal carcinoma (NPC).

**Methods**: A total of 544 patients with locoregionally advanced NPC that was newly diagnosed from January 2009 to December 2015 were included in this study. Among these patients, 251 were treated with TP induction chemotherapy followed by CCRT with cisplatin (DDP) alone (TP + DDP group), 167 were treated with TP followed by CCRT with TP (TP + TP group), and 126 were treated with docetaxel, DDP and fluorouracil (TPF) followed by CCRT with DDP alone (TPF + DDP group). Overall survival (OS), distant metastasis-free survival (DMFS), progression-free survival (PFS) and locoregional relapse-free survival (LRRFS) were analyzed using the Kaplan-Meier method and a Cox proportional hazards model.

**Results**: Survival analysis showed that the 5-year OS, PFS and DMFS rates in the TP + DDP group were significantly lower than those in the TP + TP group after propensity score matching (PSM). Multivariate analysis revealed that CCRT with TP was an independent prognostic factor for OS, PFS and DMFS. During CCRT, the incidence rates of grade 3/4 nausea/vomiting, oral mucositis, leukocytopenia and neutropenia were significantly increased in the TP + TP group compared with the TP + DDP group (all *P* < 0.05). To further explore the value of TP + TP, we performed PSM again with the TPF + DDP group. After PSM, there were 100 patients in each group. Survival analysis showed no significant differences in the 5-year OS, PFS, DMFS and LRRFS rates between the two groups. During IC and CCRT, the rate of grade 3/4 nausea/vomiting in the TPF + DDP group was higher than that in the TP+TP group (9.0% *vs.* 2.0%,* P* = 0.030; 18.0% *vs.* 8.0%, *P* = 0.036, respectively). No significant difference in the incidence of grade 3/4 hematologic toxicity was found between the two groups (all *P* > 0.05).

**Conclusion**: TP + TP can reduce the distant metastasis of locoregionally advanced NPC and improve OS compared with TP + DDP; TP + TP has the same effect as TPF + DDP and is clinically feasible.

## Introduction

Nasopharyngeal carcinoma (NPC), originating from nasopharyngeal mucosa epithelia, is a common malignant tumor of the head and neck. Patients suffering from NPC are mainly concentrated in southeast Asia, especially in South China, and there are obvious regional characteristics^1^. The morbidity of NPC is 20~30 per 100,000 people in Guangdong Province^2^. Because of a lack of specificity of the early symptoms, 70% of patients diagnosed with NPC are in advanced stages^3^, ^4^. Radiotherapy is the main treatment for NPC because the primary tumor exhibits a unique anatomical location and radiosensitivity^1^,^5^.

The intergroup 0099 trial demonstrated that concurrent chemoradiotherapy (CCRT) with high-dose cisplatin (DDP) followed by adjuvant chemotherapy (AC) significantly improved overall survival (OS) and progression-free survival (PFS) in patients with locoregionally advanced NPC, which was confirmed by several large clinical studies in an NPC endemic area^6^-^9^. Therefore, CCRT with DDP followed by AC has become the standard treatment. However, a phase Ⅲ clinical trial found that the addition of AC followed by CCRT could not improve the 5-year OS and PFS rates compared with CCRT alone (80% *vs.* 83%, *P* = 0.35; 71% *vs.* 75%, *P* = 0.45, respectively) in patients with locoregionally advanced NPC^10^. These negative results may be attributed to the fact that only 63% of patients could complete the entire course of AC. Thus, whether AC can further improve patient survival is still unknown.

Induction chemotherapy (IC) has the advantages of better tolerance and early removal of micrometastases^11^. Sun *et al*.^12^ reported that docetaxel, cisplatin and fluorouracil (TPF) followed by CCRT with DDP alone (TPF + DDP) significantly improved OS, PFS and distant metastasis-free survival (DMFS) in patients with locoregionally advanced NPC. An individual patient data (IPD) pooled analysis recruiting four clinical trials demonstrated the positive clinical value of IC^13^. Therefore, the 2018 National Comprehensive Cancer Network (NCCN) upgraded the evidence of IC to category 2A^14^. However, Hui *et al*.^15^ found that compared with CCRT alone, docetaxel and cisplatin (TP) followed by CCRT with DDP alone (TP + DDP) could not improve the 3-year PFS (hazard ratio (HR) = 0.49; 95% confidence interval (CI) 0.20-1.19; *P* = 0.12) in locoregionally advanced NPC. Therefore, further improvement of the TP + DDP regimen is needed.

Furthermore, the role of CCRT with a double-drug regimen in locoregionally advanced NPC is unknown. CCRT with a double-drug regimen in lung cancer and cervical cancer is approved and widely used in clinical practice^16^, ^17^. Radiotherapy combined with docetaxel is feasible and is widely used in non-small-cell lung cancer (NSCLC) and cervical cancer^18^, ^19^. Recently, a study demonstrated that CCRT combined with a small weekly dose of docetaxel is an effective and tolerable treatment for locoregionally advanced NPC^20^. It is not clear whether CCRT with a double-drug (docetaxel + DDP) regimen could improve survival compared with CCRT with DDP alone.

Therefore, this study aimed to explore the significance of TP-based IC followed by CCRT with a TP regimen in locoregionally advanced NPC.

## Patient selection and methods

### Patient selection

**Fig 1:**
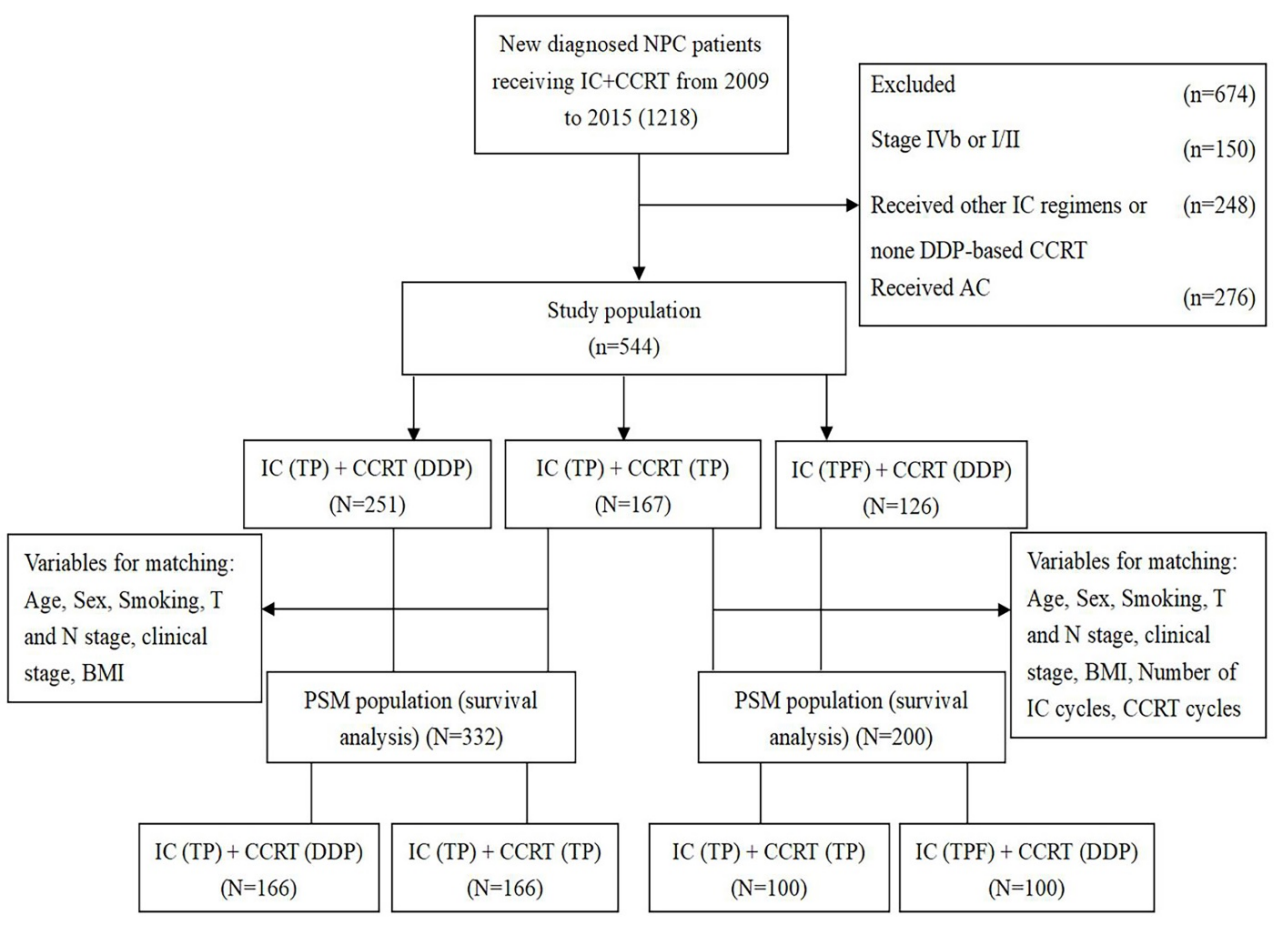
Flow chart of patient selection. IC: induction chemotherapy; CCRT: concurrent chemoradiotherapy; TP: docetaxel and cisplatin; TPF: docetaxel, cisplatin and fluorouracil; PSM: propensity score matching; DDP: cisplatin; BMI: body mass index.

This study was approved by the ethics committee of the Affiliated Cancer Hospital & Institute of Guangzhou Medical University. Clinical and imaging data were collected from 1218 patients with newly diagnosed locoregionally advanced NPC who received IC followed by CCRT (IC + CCRT) at the Department of Radiation Oncology of the Affiliated Cancer Hospital & Institute of Guangzhou Medical University from January 2009 to December 2015. The inclusion criteria were as follows: (1) patients with a pathologic diagnosis of WHO type Ⅰ, Ⅱ and Ⅲ NPC; (2) patients who underwent definitive intensity-modulated radiotherapy (IMRT); (3) patients with a performance status (PS) score between 0 and 1; (4) patients with stage Ⅲ-Ⅳa disease according to the 8^th^ edition of the Union for International Cancer Control/American Joint Committee on Cancer (UICC/AJCC) staging system; (5) patients administered CCRT with DDP or TP after TP induction chemotherapy; (6) patients who received TPF induction chemotherapy followed by CCRT with DDP alone; and (7) patients who received no adjuvant chemotherapy. The exclusion criteria were as follows: (1) previous history of other malignancies; (2) evidence of serious dysfunction of heart, lung, liver, kidney and other important organs; and (3) patients who were pregnant or nursing. After screening, 674 patients were excluded, of whom 150 were initially diagnosed with stage Ⅳb or Ⅰ/Ⅱ patients, 248 were not treated with TPF or TP induction chemotherapy regimen or DDP-based CCRT, and 276 were treated with AC. Finally, 544 patients were included in this study, and patients were classified into three treatment groups: patients in the TP + DDP group were treated with TP induction chemotherapy followed by CCRT with DDP alone (n = 251); patients in the TP + TP group were treated with TP induction chemotherapy followed by CCRT with TP (n = 167); and patients in the TPF + DDP group were treated with TPF-based IC followed by CCRT with DDP alone (n = 126). The details are shown in Figure 1.

### Induction chemotherapy

An IC regimen with TPF or TP was used in this study. The TPF regimen included docetaxel (60 mg/m^2^, d1), DDP (60 mg/m^2^, d1) and fluorouracil (600 mg/m^2^, 24 hours daily from d1~5). The TP regimen included docetaxel (75~80 mg/m^2^, d1) and DDP (80 mg/m^2^, d1). IC was administered every three weeks.

### Concurrent chemoradiotherapy

Radiotherapy is the main treatment for NPC. All patients were irradiated with IMRT. After being immobilized with head and neck thermoplastic masks while in the supine position, patients were scanned with a CT simulator. Noncontrast and contrast CT images were collected with 3 mm per slice from the vertex to 2 cm below the clavicle head. The target volumes were contoured according to the Sun Yat-sen University Cancer Center institutional treatment protocol^21^, which is in agreement with the International Commission on Radiation Units and Measurements Reports 50 and 62. The prescribed radiation dose to the gross tumor volume of nasopharyngeal tumors (GTVnx) was 70~74 Gy, and the prescribed radiation dose to the gross tumor volume of lymph nodes (GTVnd) was 68~70 Gy. The high-risk clinical target volume (CTV1) and the low-risk clinical target volume (CTV2) were prescribed 60~66 Gy and 54~56 Gy, respectively. All patients were irradiated with 30 to 32 fractions in total, once daily, Monday through Friday. Patients in the TP + DDP group or the TPF + DDP group were treated with 1~3 cycles of DDP (80~100 mg/m², d1) every 21 days during radiotherapy. Patients in the TP + TP group were given DDP (75~80 mg/m², d1) and docetaxel (75 mg/m², d1) treatment, which was repeated every 21 days for 1~3 cycles during radiotherapy.

### Follow-up

After treatment, the patients were followed every 3 months for 2 years, every 6 months from the 3^rd^ to 5^th^ years, and every year thereafter. Follow-up data were obtained from outpatient and inpatient medical records and telephone counseling. The follow-up time was calculated from the date of diagnosis to the last date of follow-up or death. OS was calculated from the date of diagnosis until the follow-up deadline or death. PFS was defined as the time from initial diagnosis to tumor progression or death; DMFS was defined as the time to tumor metastasis; and locoregional relapse-free survival (LRRFS) was defined as the time to the first locoregional recurrence. Hematologic toxicity, liver and kidney function, and oral mucosal responses were graded according to the National Cancer Institute Common Terminology Criteria for Adverse Events Version 4.0 (NCI-CTCAE 4.0)^22^.

### Data analysis

SPSS 25.0 (Chicago, IL, USA) was used for all data analysis. Clinical features and toxicities were analyzed using the chi-square test or Fisher's test. In this study, propensity score matching (PSM) was used to exclude observable confounding factors^23^, and covariates were included in the PSM analysis, including age, sex, smoking history, T stage, N stage, clinical stage, pretreatment body mass index (BMI), and number of IC and CCRT cycles. The survival outcomes of the two groups' PSM cohort were analyzed by the Kaplan-Meier method. Multivariate analysis was performed using a Cox proportional hazards regression model, and the analyzed variables included T stage, N stage, clinical stage, age, smoking history, and number of IC courses, CCRT cycles and CCRT regimens. All statistical analyses were defined with significance level of *P* < 0.05. All trials were bilateral, and the results of the multivariate analysis were expressed by both the HR and the 95% CI.

## Results

Before PSM, there were 418 patients in total, with 251 patients in the TP + DDP group and 167 patients in the TP + TP group. After PSM, 332 patients were included, with 166 patients in each group. The baseline clinical characteristics of the TP + DDP and TP + TP groups are shown in Table 1.

**Fig 2:**
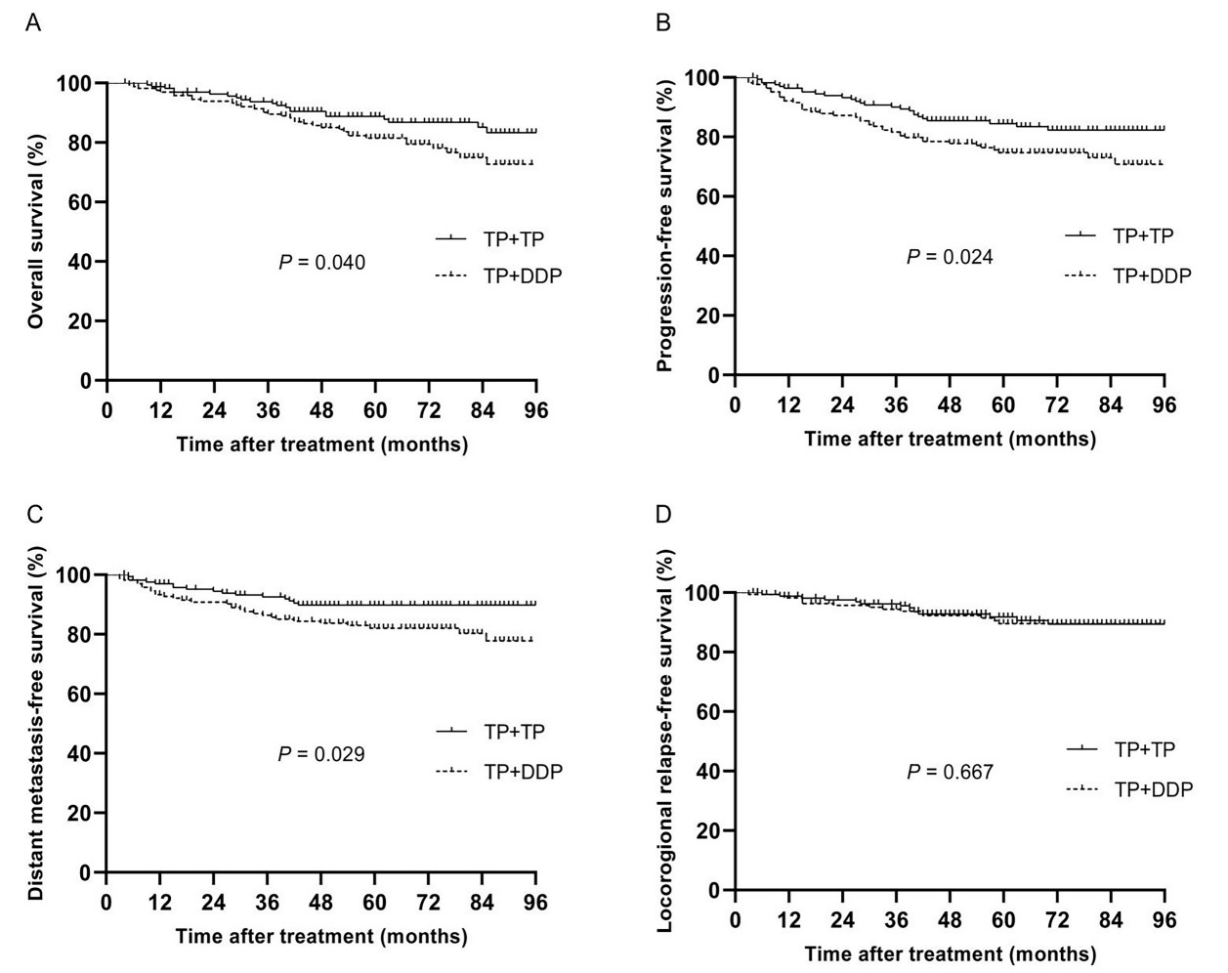
Kaplan-Meier analysis of survival between TP + DDP and TP + TP groups after propensity score matching. A: Overall survival; B: Progression-free survival; C: Distant metastasis-free survival; D: Locoregional relapse-free survival.

The median follow-up time of 332 patients was 64 months (4~114 months), and the 5-year OS, PFS, DMFS and LRRFS rates were 84.9%, 79.4%, 85.8% and 90.6%, respectively. The 5-year OS, PFS, DMFS and LRRFS rates in the TP + DDP group *vs.* TP + TP group were 81.5% *vs.* 88.8%, 74.7% *vs.* 84.5%, 82.0% *vs.* 89.8%, and 91.8% *vs.* 89.6%, respectively (*P* = 0.040, Fig. 2A; *P* = 0.024, Fig. 2B; *P* = 0.029, Fig. 2C; and *P* = 0.667, Fig. 2D, respectively).

As shown in Table 2, Cox regression multivariate analysis was used to adjust for various prognostic factors. Grouping factors of TP + TP significantly improved the 5-year OS (HR, 0.563; 95% CI 0.325 to 0.974; *P* = 0.048), PFS (HR, 0.585; 95% CI 0.359 to 0.953; *P* = 0.031) and DMFS (HR, 0.523; 95% CI 0.285 to 0.961; *P* = 0.037). Clinical staging was an independent prognostic factor for OS, PFS, DMFS and LRRFS (all *P* < 0.05). N staging and IC cycles was an independent prognostic factor for OS, PFS and DMFS (all *P* < 0.05).

**Fig 3:**
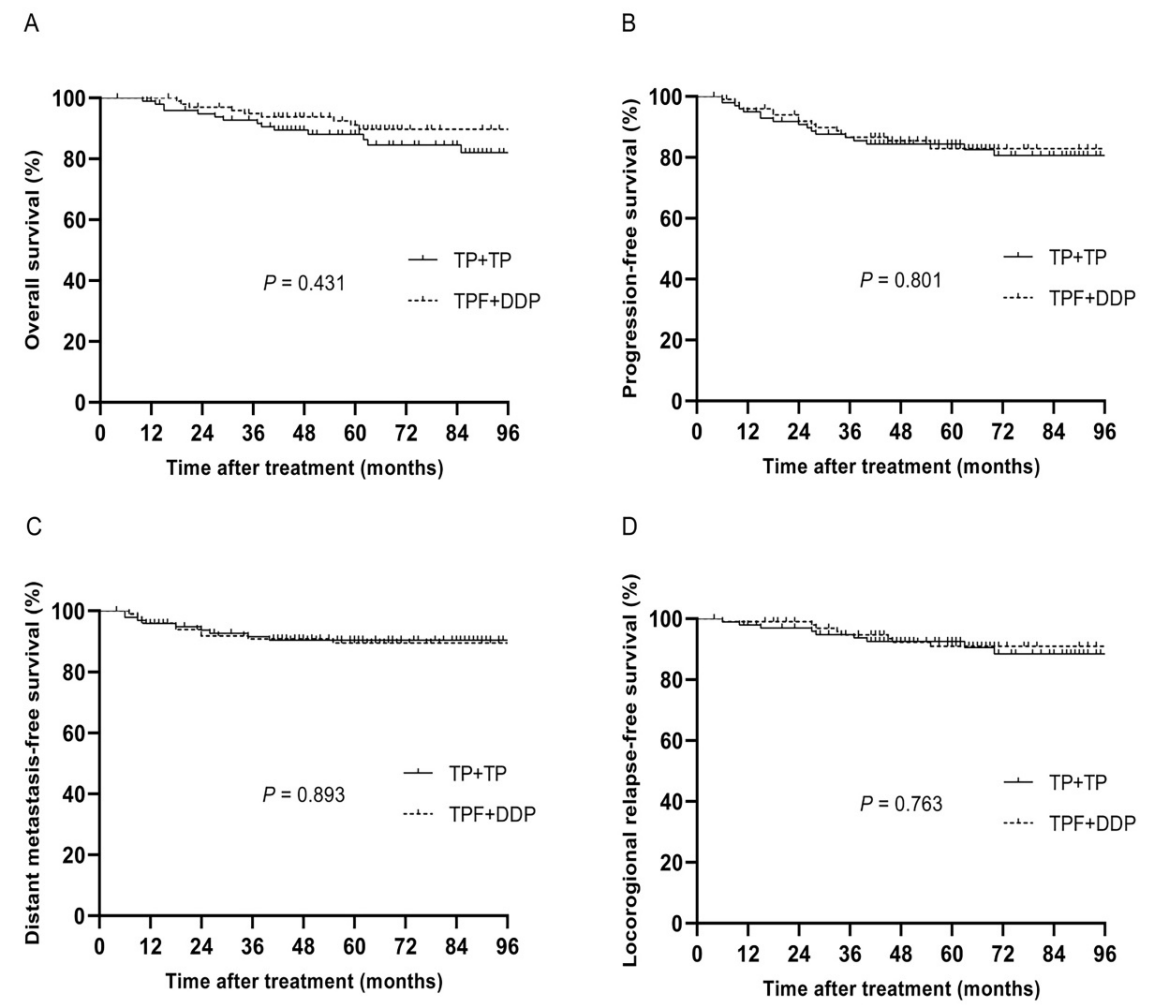
Kaplan-Meier analysis of survival between TPF + DDP and TP + TP groups after propensity score matching. A: Overall survival; B: Progression-free survival; C: Distant metastasis-free survival; D: Locoregional relapse-free survival.

Before PSM, 293 patients were divided into two groups, with 126 patients in the TPF + DDP group and 167 patients in the TP + TP group. After PSM, 200 patients were identified, and there were 100 patients in each cohort. The specific baseline characteristics are shown in Table 3. The median follow-up time of these 200 patients was 63 months (4~113 months), and the 5-year OS, PFS, DMFS and LRRFS rates were 88.8%, 83.5%, 89.9% and 91.6%, respectively. The 5-year OS, PFS, DMFS and LRRFS rates in the TPF + DDP group *vs.* TP + TP group were 88.0% *vs.* 89.7%, 84.4% *vs.* 82.9%, 89.5% *vs.* 90.5%, and 92.6% *vs.* 91.0%, respectively (*P* = 0.431, Fig. 3A; *P* = 0.801, Fig. 3B; *P* = 0.893, Fig. 3C; and *P* = 0.763, Fig. 3D, respectively).

Multivariate analysis demonstrated that there were no significant differences between the TPF + DDP group and the TP + TP group in 5-year OS, PFS, DMFS and LRRFS. Clinical staging was an independent factor for OS, PFS and DMFS (Table 4).

### Toxic effects

Table 5 shows patients' toxicities during IC in the PSM cohort. There were no differences in hematologic toxicities or nonhematologic toxicities between the TP + DDP group and the TP + TP group (all *P* > 0.05). The hematologic toxicities in the TPF + DDP group were similar to those in the TP + TP group (all *P* > 0.05). In terms of nonhematologic toxicities, the acute adverse events of grade 3/4 nausea/vomiting in the TPF + DDP group were higher than those in the TP + TP group (*P* = 0.033). However, hypoalbuminemia, liver or kidney function did not show significant differences between the two groups (all *P* > 0.05).

In the PSM cohort, patients' toxicities during CCRT are shown in Table 6. The rates of grade 3/4 leukocytopenia and neutropenia in the TP + TP group occurred in 44.0% and 39.8% of patients, respectively, which were significantly higher than those in the TP + DDP group (19.9% and 16.3%, respectively; *P* < 0.001). In terms of nonhematologic toxicities, the rates of grade 3/4 oral mucosal and nausea/vomiting reactions in the TP + TP group were significantly higher than those in the TP + DDP group (*P* < 0.001 and *P* = 0.035, respectively). The rates of acute adverse events of grade 3/4 nausea/vomiting in the TPF + DDP group were higher than those in the TP + TP group (18.0% *vs*. 8.0%; *P* = 0.036). However, there were no significant differences in other adverse events (all *P* > 0.05).

## Discussion

This study is the first to retrospectively explore the long-term efficacy and toxicities of CCRT with double-drug chemotherapy in locoregionally advanced NPC using PSM. First, under the condition of the same IC regimen, TP + TP group had superior OS, PFS and DMFS over those in the TP + DDP group in locoregionally advanced NPC. Second, we found that the 5-year OS, PFS, DMFS and LRRFS rates were similar between the TP + TP group and the TPF + DDP group, but the rates of acute adverse events of grade 3/4 nausea/vomiting responses were higher in the TPF + DDP group than in the TP + TP group during the IC and CCRT period; this was the main clinical benefit in the TP + TP group.

Radiotherapy combined with DDP in locoregionally advanced NPC has been repeatedly demonstrated^7^-^9^. However, the distant metastasis rate still reached 30% in locoregionally advanced NPC^24^. Therefore, the addition of AC or IC has been used to decrease the distant metastasis rate in locoregionally advanced NPC. However, the significance of AC is still controversial in locoregionally advanced NPC^25^. Furthermore, compared with AC, IC can improve tolerance and eradicate micrometastases^11^. At present, several prospective studies have demonstrated the clinical efficacy of IC. Furthermore, an IPD pooled analysis including four clinical trials from endemic NPC regions confirmed that the IC + CCRT regimen could improve the 5-year OS (HR = 0.75; 95% CI 0.90 to 0.51) and DMFS (HR = 0.68, 95% CI 0.57 to 0.99)^13^. Hui *et al*.^15^ showed the feasibility of IC with TP in locoregionally advanced NPC. However, the efficacy of the TP + DDP regimen was low, and the evidence level was only Category 2B according to the NCCN guidelines^14^. Therefore, the TP + DDP regimen needs further investigation of the treatment of locally advanced NPC. Some studies demonstrated that the objective remission rate (ORR) of CCRT with the TP regimen was 100% in locoregionally advanced cervical cancer^26^. The RTOG 9410 reported that the 5-year OS in stage Ⅲ NSCLC with double-drug regimen CCRT was 16%, and this has become the standard clinical treatment in stage Ⅲ NSCLC^16^. The clinical effect of CCRT with a double-drug regimen in locoregionally advanced NPC is worth exploration.

Docetaxel is used in the treatment of many solid tumors by blocking mitosis to achieve an antitumor effect^27^. A retrospective study demonstrated that docetaxel combined with radiotherapy had a similar clinical effect as DDP combined with radiotherapy in locoregionally advanced NPC^20^. Docetaxel showed great drug activity and an improved radiotherapy sensitization effect in head and neck cancers^27^. Therefore, whether the combination of DDP plus docetaxel concurrent with radiotherapy can further improve the survival rate of patients with locoregionally advanced NPC needs to be investigated. A phase Ⅱ prospective study evaluated the short-term efficacy of TP-based IC followed by CCRT with TP regimen versus DDP alone in locoregionally advanced NPC and showed that the complete response (CR) rate of patients was similar in the two groups (93.3% *vs.* 96.3%, respectively)^28^. However, in our study, the long-term efficacy revealed that CCRT with a TP double-drug regimen could significantly improve patients' 5-year OS, PFS and DMFS compared with CCRT with DDP alone under the condition of the same IC regimen. Furthermore, the TP double-drug regimen was likely to improve OS by reducing the distant metastasis.

In recent years, the TPF + DDP regimen has become a category Ⅰ recommendation for Epstein-Barr virus (EBV)-related NPC patients in the NCCN guidelines based on clinical trial results^12^, ^13^. The TPF + DDP regimen had a high clinical value at the price of severe side effects, including hematologic toxicities and nonhematologic toxicities. Therefore, the optimal treatment regimen of locoregionally advanced NPC still needs further investigation. This study found that the 5-year OS, PFS, DMFS and LRRFS rates of the TP + TP group and the TPF + DDP group were not significantly different. However, the rate of grade 3/4 nausea/vomiting in the TP + TP group was lower than that in the TPF + DDP group during the IC and CCRT periods, which was the main clinical benefit in the TP + TP group.

Xie *et al*.^28^ reported that grade 3/4 hematologic toxicities in the TP + TP group were higher than those in the TP + DDP group during CCRT, which was consistent with the results of our study. There was no significant difference in grade 3/4 oral mucositis between the two groups in Xie's study^28^, which showed 78.6% in the TP + TP group and 76.0% in the TP + DDP group. However, our study demonstrated that patients in the TP + TP group had higher rates of oral mucositis than those in the TP + DDP group (36.0% *vs.* 16.9%; *P* < 0.001). The different results in this study and Xie's study may be related to the different radiotherapy techniques. In the era of IMRT, the rate of oral mucositis has significantly decreased^29^. The application of fluorouracil in IC and high-dose DDP (100 mg/m^2^) in CCRT may result in the higher rates of grade 3/4 nausea/vomiting in the TPF + DDP group than in the TP + TP group.

At present, few studies have been published on the clinical value of CCRT with a double-drug regimen for locoregionally advanced NPC. This study confirmed the positive clinical value of CCRT with a TP double-drug regimen, which significantly decreased the rate of distant metastasis and improved the OS. The reason for this conclusion may be attributed to the fact that CCRT with a double-drug regimen was superior to CCRT with a single-drug regimen in preventing lymph node recurrence and distant metastasis. In this study, although the hematologic toxicities of the TP regimen were worse than those of the DDP alone regimen during CCRT, they were under clinical control. In summary, it is feasible to apply CCRT with a TP regimen in clinic. Furthermore, although the 5-year OS in the TP + TP group was similar to that in the TPF + DDP group, there was lower grade 3/4 nausea/vomiting toxicity in the TP + TP group. Therefore, TP induction chemotherapy followed by CCRT with a TP regimen could be adopted for the treatment of locoregionally advanced NPC.

This study has some limitations. Because biases are inevitable in a retrospective study, we utilized PSM to exclude observable confounding factors. Furthermore, unknown potential confounding factors along with some lost cases are also issues. Additionally, some toxicities in some outpatients were not recorded adequately. Therefore, the results of this study need to be confirmed by a large prospective clinical study.

## Conclusion

In patients with locoregionally advanced NPC treated with TP-based IC, compared with CCRT with DDP alone, the following CCRT with TP regimen decreased the rate of distant metastasis and improved OS; thus, we can draw the conclusion that CCRT with double-drug chemotherapy improved survival. Furthermore, we found that the 5-year OS, PFS, DMFS and LRRFS rates were similar between the TP + TP group and the TPF + DDP group, but the rates of acute adverse events of grade 3/4 nausea/vomiting responses were higher in the TPF + DDP group than in the TP + TP group during the IC and CCRT period; this was the main clinical benefit in the TP + TP group. Therefore, it might be optimal to treat patients with locoregionally advanced NPC with TP-based IC followed by CCRT with TP.

## Figures and Tables

**Table 1:** Characteristics of patients in the TP + TP and TP + DDP groups before and after propensity score matching

Item	Entire cohort (%)					Propensity score-matched cohort (%)			
	TP+DDP	TP+TP	*P*			TP+DDP	TP+TP	*P*	
Total	251 (60.0)	167 (40.0)				166 (50.0)	166 (50.0)		
Age (y)			0.765					0.509	
< 46	118 (47.0)	81 (48.5)				74 (44.6)	80 (48.2)		
≥ 46	133 (53.0)	86 (51.5)				92 (55.4)	86 (51.8)		
Gender			0.672					0.536	
Male	174 (69.3)	119 (71.3)				124 (74.7)	119 (71.7)		
Female	77 (30.7)	48 (28.7)				42 (25.3)	47 (28.3)		
T stage			<0.001					0.110	
T1-2	41 (16.3)	53 (31.7)				39 (23.5)	52 (31.3)		
T3-4	210 (83.7)	114 (68.3)				127 (76.5)	114 (68.7)		
N stage			0.738					1.000	
N0-1	79 (31.5)	54 (32.3)				54 (32.5)	54 (32.5)		
N2-3	172 (68.5)	113 (67.7)				112 (67.5)	112 (68.5)		
Clinical stage			0.449					0.720	
III	167 (66.5)	117 (70.1)				114 (68.7)	117 (70.5)		
IVa	84 (33.5)	50 (29.9)				52 (31.3)	49 (29.5)		
Smoking			0.673					0.659	
Yes	103 (41.0)	72 (36.5)				76 (45.8)	72 (43.4)		
No	148 (59.0)	95 (56.9)				90 (54.2)	94 (56.6)		
CCRT cycles			0.166					0.667	
< 2	56 (22.3)	28 (16.8)				31 (18.7)	28 (16.9)		
≥ 2	195 (77.7)	139 (83.2)				135 (81.3)	138 (83.1)		
IC cycles			0.721					0.158	
< 2	63 (25.1)	47 (28.1)				59 (35.5)	47 (28.3)		
≥ 2	188 (74.9)	120 (71.9)				107 (64.5)	119 (71.7)		
BMI (kg/m²)			0.977					0.859	
< 18	11 (4.4)	8 (4.8)				6 (3.6)	8 (4.8)		
18-24	164 (65.3)	108 (64.7)				109 (65.7)	107 (64.5)		
> 24	76 (30.3)	51 (30.5)				51 (30.7)	51 (30.7)		
Histology			0.882					0.598	
I	1 (0.4)	1 (0.6)				1 (0.6)	1 (0.6)		
II	6 (2.4)	3 (1.8)				6 (3.6)	3 (1.8)		
III	244 (97.2)	163 (97.6)				159 (95.8)	162 (97.6)		

IC: induction chemotherapy; CCRT: concurrent chemoradiotherapy; TP: docetaxel and cisplatin; DDP: cisplatin; BMI: body mass index.

**Table 2:** Multivariate analysis of prognostic factors in 332 patients with NPC after propensity score matching

Endpoint	Variable	Hazard ratio	95% CI	*P*
OS	Group (TP+TP *vs.* TPF+DDP)	0.563	(0.325-0.974)	0.048
	N stage	2.281	(1.189-4.378)	0.013
	Clinical stage (III *vs.* IVa)	2.699	(1.589-4.585)	0.000
	IC cycles (< 2 cycles *vs.* ≥ 2 cycles)	0.544	(0.318-0.929)	0.026
PFS	Group (TP+TP *vs.* TPF+DDP)	0.585	(0.359-0.953)	0.031
	N stage	2.297	(1.266-4.165)	0.006
	Clinical stage (III *vs.* IVa)	2.418	(1.496-3.908)	0.000
	IC cycles (< 2 cycles *vs.* ≥ 2 cycles)	0.558	(0.344-0.905)	0.018
DMFS	Group (TP+TP *vs.* TPF+DDP)	0.523	(0.285-0.961)	0.037
	N stage	2.740	(1.271-5.908)	0.010
	Clinical stage (III *vs.* IVa)	2.694	(1.494-4.855)	0.001
	IC cycles (< 2 cycles *vs.* ≥ 2 cycles)	0.418	(0.232-0.752)	0.004
LRRFS	Clinical stage (III *vs.* IVa)	2.154	(1.051-4.417)	0.036

IC: induction chemotherapy; CCRT: concurrent chemoradiotherapy; TP: docetaxel and cisplatin; DDP: cisplatin; HR: hazard ratio.

**Table 3:** Characteristics of patients between the TPF + DDP group and TP+TP group before and after propensity score matching.

Item	Entire cohort (%)					Propensity-score matched cohort (%)			
	TPF+DDP	TP+TP	*P*			TPF+DDP	TP+TP	*P*	
Total	126 (43.0)	167 (57.0)				100 (50.0)	100 (50.0)		
Age			0.905					0.887	
< 46	62 (49.2)	81 (48.5)				51 (51.0)	52 (52.0)		
≥ 46	64 (50.8)	86 (51.5)				49 (49.0)	48 (48.0)		
Gender			0.114					0.626	
Male	100 (79.4)	119 (71.3)				76 (76.0)	73 (73.0)		
Female	26 (20.6)	48 (28.7)				24 (24.0)	27 (27.0)		
T stage			0.181					1.000	
T1-2	31 (24.6)	53 (31.7)				27 (27.0)	27 (27.0)		
T3-4	95 (75.4)	114 (68.3)				73 (73.0)	73 (73.0)		
N stage			0.026					0.154	
N0-1	26 (20.6)	54 (32.3)				23 (23.0)	32 (32.0)		
N2-3	100 (79.4)	113 (67.7)				77 (77.0)	68 (68.0)		
Clinical stage			0.003					0.884	
III	67 (53.2)	117 (70.1)				62 (62.0)	61 (61.0)		
IVa	59 (46.8)	50 (29.9)				38 (38.0)	39 (39.0)		
Smoking			0.857					0.776	
Yes	53 (42.1)	72 (36.5)				43 (43.0)	45 (45.0)		
No	73 (57.9)	95 (56.9)				57 (57.0)	55 (55.0)		
CCRT cycles			0.983					0.171	
< 2	18 (14.3)	28 (16.8)				12 (12.0)	19 (19.0)		
≥ 2	108 (85.7)	139 (83.2)				88 (88.0)	81 (81.0)		
IC cycles			<0.001					0.637	
< 2	11 (8.7)	47 (28.1)				11 (11.0)	9 (9.0)		
≥ 2	115 (91.3)	120 (71.9)				89 (89.0)	91 (91.0)		
BMI (kg/m²)			0.785					0.752	
< 18	5 (4.0)	8 (4.8)				5 (5.0)	5 (5.0)		
18-24	78 (61.9)	108 (64.7)				58 (58.0)	63 (63.0)		
> 24	43 (34.1)	51 (30.5)				37 (37.0)	32 (32.0)		
Histology			0.751					0.359	
I	1 (0.8)	1 (0.6)				0 (0.0)	1 (1.0)		
II	1 (0.8)	3 (1.8)				1 (1.0)	3 (3.0)		
III	124 (98.4)	163 (97.6)				99 (99.0)	96 (96.0)		

IC: induction chemotherapy; CCRT: concurrent chemoradiotherapy; TP: docetaxel and cisplatin; TPF: docetaxel, cisplatin and fluorouracil; DDP: cisplatin; BMI: body mass index.

**Table 4:** Multivariate analyses of prognostic factors in 200 patients with nasopharyngeal carcinoma after propensity score matching.

Endpoint	Variable	Hazard ratio	95% CI	*P*
OS	Clinical stage (III *vs.* IVa)	2.646	(1.179-5.939)	0.018
	smoking (yes *vs.* no)	2.310	(1.033-5.169)	0.042
PFS	Clinical stage (III *vs.* IVa)	2.618	(1.302-5.264)	0.007
DMFS	Clinical stage (III *vs.* IVa)	4.288	(1.529-12.02)	0.006
LRRFS	CCRT (< 2 cycles *vs.* ≥ 2 cycles)	0.310	(0.115-0.839)	0.021

CCRT: concurrent chemoradiotherapy; HR: hazard ratio.

**Table 5:** Adverse events during IC after propensity score matching

Adverse events	TP+DDP (case%)		TP+TP (case%)		*P*		TPF+DDP regimen (case%)		TP+TP regimen (case%)		*P*
	Grade 0-2	Grade 3-4	Grade 0-2	Grade 3-4			Grade 0-2	Grade 3-4	Grade 0-2	Grade 3-4	
**Hematologic**											
Leukocytopenia	155 (93.4)	11 (6.6)	150 (90.4)	16 (9.6)	0.315		93 (93.0)	7 (7.0)	90 (90.0)	10 (10.0)	0.447
Neutropenia	152 (91.6)	14 (8.4)	147 (88.6)	19 (11.4)	0.359		85 (85.0)	15 (15.0)	89 (89.0)	11 (11.0)	0.400
Anemia	165 (99.4)	1 (0.6)	165 (99.4)	1 (0.6)	1.000		99 (99.0)	1 (1.0)	99 (99.0)	1 (1.0)	1.000
Thrombocytopenia	165 (99.4)	1 (0.6)	166 (100)	0 (0)	1.000		99 (99.0)	1 (1.0)	100 (100)	0 (0)	1.000
**Nonhematologic**											
Liver function	166 (100)	0 (0)	166 (100)	0 (0)	1.000		100 (100)	0 (0)	100 (100)	0 (0)	1.000
Renal function	166 (100)	0 (0)	166 (100)	0 (0)	1.000		100 (100)	0 (0)	100 (100)	0 (0)	1.000
Hypoalbuminemia	166 (100)	0 (0)	166 (100)	0 (0)	1.000		100 (100)	0 (0)	100 (100)	0 (0)	1.000
Nausea/vomiting	162 (97.6)	4 (2.4)	163 (98.2)	3 (1.8)	0.702		90 (90.0)	10 (10.0)	98 (98.0)	2 (2.0)	0.033

IC: induction chemotherapy; TP: docetaxel and cisplatin; TPF: docetaxel, cisplatin and fluorouracil; DDP: cisplatin. Adverse events were graded according to the National Cancer Institute Common Toxicity Criteria, version 4.0.

**Table 6:** Adverse events during CCRT after propensity score-matched

Adverse event	TP+DDP regimen (case%)		TP+TP regimen (case%)		*P*-value		TPF+DDP regimen (case%)		TP+TP regimen (case%)		*P*-value
	Grade 0-2	Grade 3-4	Grade 0-2	Grade 3-4			Grade 0-2	Grade 3-4	Grade 0-2	Grade 3-4	
**Hematologic**											
Leukocytopenia	133 (80.1)	33 (19.9)	93 (56.0)	73 (44.0)	<0.001		73 (73.0)	37 (37.0)	58 (58.0)	42 (42.0)	0.211
Neutropenia	139 (83.7)	27 (16.3)	100 (60.2)	66 (39.8)	<0.001		77 (77.0)	33 (33.0)	62 (62.0)	38 (38.0)	0.460
Anemia	165 (99.4)	1 (0.6)	163 (98.2)	3 (1.8)	0.623		93 (93.0)	7 (7.0)	97 (97.0)	3 (3.0)	0.331
Thrombocytopenia	165 (99.4)	1 (0.6)	165 (99.4)	1 (0.6)	1.000		95 (95.0)	5 (5.0)	99 (99.0)	1 (1.0)	0.212
**Nonhematologic**											
Liver function	162 (97.6)	4 (2.4)	164 (98.8)	2 (1.2)	0.685		100 (100)	0 (0)	100 (100)	0 (0.0)	1.000
Renal function	166 (100)	0 (0)	166 (100)	0 (0)	1.000		100 (100)	0 (0)	100 (100)	0 (0.0)	1.000
Hypoalbuminemia	166 (100)	0 (0)	166 (100)	0 (0)	1.000		100 (100)	0 (0)	100 (100)	0 (0.0)	1.000
Oral mucositis	138 (83.1)	28 (16.9)	106 (63.9)	60 (36.1)	<0.001		77 (77.0)	23 (23.0)	67 (67.0)	33 (33.0)	0.115
Nausea/vomiting	161 (97.0)	5 (3.0)	151 (91.0)	15 (9.0)	0.035		82 (82.0)	18 (18.0)	92 (92.0)	8 (8.0)	0.036

CCRT: concurrent chemoradiotherapy; TP: docetaxel and cisplatin; TPF: docetaxel, cisplatin and fluorouracil; DDP: cisplatin. Adverse events were graded according to the National Cancer Institute Common Toxicity Criteria, version 4.0.

## Abbreviations

NPC: nasopharyngeal carcinoma
IC: induction chemotherapy
IMRT: intensity-modulated radiotherapy
CCRT: concurrent chemoradiotherapy
PSM: propensity score matching
EBV: Epstein-Barr virus
TP: docetaxel plus cisplatin
TPF: docetaxel, cisplatin and fluorouracil
DDP: cisplatin
NCI-CTCAE 4.0: National Cancer Institute Common Terminology Criteria for Adverse Events Version 4.0
GTV: gross tumor volume
CTV: clinical tumor volume
OS: overall survival
DMFS: distant metastasis-free survival
PFS: progression-free survival
LRRFS: locoregional relapse-free survival
HR: hazard ratio
BMI: body mass index
CI: confidence interval
WHO: World Health Organization
NCCN: National Comprehensive Cancer Network
